# Polycyclic Aromatic Hydrocarbons (PAHs) Contamination in *Chrysichthys nigrodigitatus* Lacépède, 1803 from Lake Togo-Lagoon of Aného, Togo: Possible Human Health Risk Suitable to Their Consumption

**DOI:** 10.3390/ijerph20031666

**Published:** 2023-01-17

**Authors:** Kamilou Ouro-Sama, Gnon Tanouayi, Hodabalo Dheoulaba Solitoke, Narcis Barsan, Emilian Mosnegutu, Tchaa Esso-Essinam Badassan, Sadikou Agbere, Koudjo Adje, Valentin Nedeff, Kissao Gnandi

**Affiliations:** 1 Laboratoire de Gestion, Traitement et Valorisation des Déchets, Faculté des Sciences, Département de Géologie et Environnement, Université de Lomé, Lomé BP 1515, Togo; 2 Faculty of Engineering, “Vasile Alecsandri” University of Bacau, 157 Calea Marasesti, 600115 Bacau, Romania; 3 “Gheorghe Ionescu Sisesti” Academy of Agricultural and Forestry Sciences, 6 Marasti Blvd., 011464 Bucharest, Romania

**Keywords:** contamination, *Chrysichthys nigrodigitatus*, polycyclic aromatic hydrocarbons, health risk, Lake Togo

## Abstract

The Lake Togo-Lagoon of Aného is located in the coastal zone where phosphorite mining is carried out. This mining discharges all kinds of waste such as fuel oil into the surrounding environment without prior treatment. Moreover, the hydrosystem receives runoff and river inputs after having crossed and leached from mining and urban soils. This study aims to determine the polycyclic aromatic hydrocarbons (PAHs) contamination in commercially consumed fish species (*Chrysichthys nigrodigitatus*) from that hydrosystem and the associated health risks for consumers. For that, fish sample collection was performed during the dry season. Afterwards, their muscles, gills, and livers were cut and 12 PAHs were analyzed using gas chromatography coupled with a mass spectrometer (GC-MS). The total PAHs (tPAHs) concentrations in fish tissues ranged from 5.24 to 48.40 µg/kg with average concentration of 14.51 ± 8.95 µg/kg in muscles, from 5.90 to 28.20 µg/kg averaging 14.90 ± 5.19 µg/kg in gills, and from 43.20 to 149.00 µg/kg with an average of 80.74 ± 27.08 µg/kg in livers. The average concentrations of low molecular weight PAHs (LMW PAHs) were 10.82 ± 9.61 µg/kg in muscles, 8.25 ± 5.43 µg/kg in gills, and 47.97 ± 22.56 µg/kg in livers whereas those of high molecular weight of PAHs (HMW PAHs) were 4.10 ± 2.14 µg/kg in muscles, 7.98 ± 3.96 µg/kg in gills, and 32.77 ± 8.66 µg/kg in livers. An overall trend of decreasing concentrations with increasing fish size classes was observed for some PAHs (Naphtalene, Pyrene in muscles, for Pyrene, Benzo(a)Anthracene, Chrysene in gills and for Naphtalene, Phenanthrene in livers). The PAHs in these fish might have pyrogenic and petrogenic sources, with the dominance of pyrogenic, and all of the total hazard quotients (THQ) are less than 1. This situation must not be neglected for better planning sustainable management of the target ecosystem.

## 1. Introduction

Nowadays, the more vulnerability of the aquatic environment to several sources of pollutants is well known. It can be considered to be a veritable tank for most of the environmental contaminants which can accumulate in several compartments of the aquatic ecosystem, such as living beings [1,2,3]. Among these pollutants, there are polycyclic aromatic hydrocarbons (PAHs) which have several sources in the environment, such as anthropogenic sources, concerning the high temperature pyrolysis of fats and oils, or the combustion of organic compounds, e.g., tobacco, fossil fuel, grilled meat, waste, coal burning etc., often [4,5,6] from industries, automobile exhaust fumes, houses heating, combustion of biomass etc. [6,7,8]. Due to their relative chemical stability and non-biodegradability, PAHs are very persistent and ubiquitous in the environment and have a high tendency to accumulate in food chains, leading to human exposure. These characteristics classify them as very significant pollutants for environmental concern [6,9,10].

During recent decades, PAHs which are mutagenic and carcinogenic have been considered hazardous environmental pollutants. Consequently, they have received much attention due to their potential adverse on human health and ecosystem impacts [6,11,12]. Human exposure to these pollutants may cause toxic effects, such as mutagenesis, birth defects, and cancers etc. [6,13]. Malformations of embryo and larvae, growth reduction, DNA damage, endocrine alteration, and other toxic effects caused by PAHs have also been observed in marine organisms [6,14,15,16]. The major pathway of fish and other aquatic organisms’ exposure to PAHs would be the ingestion of contaminated food and diffusion phenomenon of the molecules present in the surrounding water through their gills and skin [10,17]. The fatty tissues of fish are the place of predilection for PAHs accumulation due to their lipophilic nature and high chemical stability [6,18]. Therefore, fishes are good indicators of pollution in aquatic ecosystems and have been widely used for environmental monitoring [19,20,21].

A large part of the world’s population depends on seafood, especially fish, to satisfy their nutritional requirements. Indeed, they are widely consumed as an important source of protein, energy, vitamins, polyunsaturated fatty acids, and minerals, which are known for their health benefits [1,22,23,24]. However, polluted aquatic organisms may pose significant risk to human health [1,25,26,27]. Food consumption has been identified as an important pathway for human exposure to many contaminants including PAHs. Thus, PAHs contamination of widely consumed fish species may have serious public health issues.

In Togo, the Lake Togo-Lagoon of Aného complex is a coastal located hydrosystem and in the phosphorite mining and treatment area. These activities discharge several kinds of waste into the coastal zone without any prior treatment. Thus, uncontrolled rejection of waste, such as the mismanagement of used oil from machinery maintenance and fuel used for furnace heating, can be noted. Furthermore, the hydrosystem receives runoff and river inputs after leaching from urban, agricultural, and mining soils as well as atmospheric deposition of particles from automobile exhaust gases. The species *Chrysichthys nigrodigitatus* is potentially exposed to the bioaccumulation of several kinds of pollutants due to its high trophic level, diet composition, and demersal habitat especially on the muddy bottom [28,29]. However, it has an important ecological role and presents valuable economic, nutritional and aquaculture interest in West African countries [30,31,32,33]. Like in other Sub-Saharan African countries, this species is very appreciated in Togo, where it contributes to the socio-economic well-being and food security for local populations who are mainly fishermen. However, there is no study regarding fish contamination by PAHs in Togo. Accordingly, there is no information on the health risks associated with the consumption of fish contaminated by PAHs in the country. The present study aims to determine PAH concentrations in commercially consumed fish species (*Chrysichthys nigrodigitatus*) from the Lake Togo-Lagoon of the Aného hydrosystem and to assess the associated human health risks for consumers.

## 2. Materials and Methods

### 2.1. Study Area

**Figure 1:**
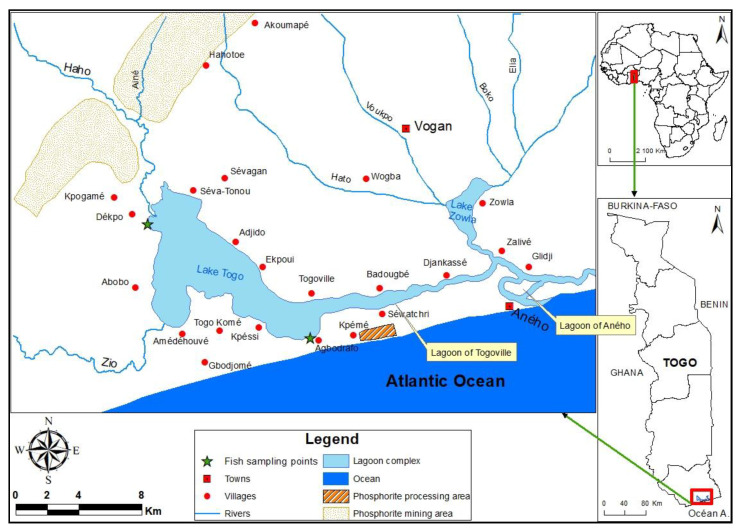
Location map showing sampling sites.

The Hydrosystem Lake Togo-Lagoon of Aného located between latitudes North 6°17′37″ and 6°14′38″ and longitudes East 1°23′33″ and 1°37′38″. It is composed of Lake Togo with an area of 46 km^2^, the lagoon of Togoville whose length and width are respectively 13 km and 150–900 m, and the Aného lagoon consisting of a narrow channel. The hydrosystem mainly receives inputs from Zio and Haho River [34]. The hydrosystem communicates with the sea at Aného (Figure 1). Phosphorite mining take place in its watershed with the discharge of several kinds of untreated mining waste. This watershed enjoys a subequatorial climate with two main seasons (rainy and dry) alternated by two small seasons (rainy and dry). The main economic activities of the populations around the hydrosystem are dominated by fishery. Other activities, e.g., agriculture and livestock cultivation, can be noted in the study area.

### 2.2. Sample Collection, Extraction and Cleanup

The fish samples were collected during the dry season in the hydrosystem at Dékpo and Agbodrafo in collaboration with the fishermen using passive collection method according to [35]. Indeed, fishes were cut using gillnets, single lines, longlines, and traps. They were individually wrapped with aluminum foil and put in polyethylene bags because of the photo-degradability of PAHs. These samples were then transported to the laboratory, in a cooler containing ice cubes, where they were washed with tap water and then rinsed with distilled water. After that, they were measured (total length), weighted (total weight) and dissected. The muscles, gills and livers that were taken and stored in the freezer at −20 °C [1,36]. A total of 30 composites of each fish organ (muscle, liver, gills) were made from groups of 4–6 fishes according to the method of Pascal et al. [37] which states that the smallest fish size in each group must not be less than 75% of the biggest one. The number of fish in each group varied according to the amount of organ necessary for analyses. Each composite sample of fish organs was ground and homogenized using a grinder (Retsch Grindomix GM 200), labelled, placed in amber glass vials, and stored at −20 °C.

The quick, effective, cheap, easy, rugged, safe (QuEChERS) methodology was used to extract PAHs from fish samples as it has been previously used by other authors for the analysis of PAHs [8,38]. Indeed, 10 g of ground and homogenized fresh samples were mixed with distilled water (15 mL) in QuEChERS tubes. After that, 15 mL of acetonitrile (CH_3_CN) and 0.1 mL of surrogate standard containing p-terphenyl-d14 (100 μg/mL), were added to each extraction tube and shaken for one-minute. Following that, 8 g of magnesium sulfate (MgSO_4_) and 2 g of sodium chloride (NaCl) were added to the QuEChERS tubes containing the previous mixtures. They were shaken vigorously for 1 min before centrifuging the extracts at 3500 rpm for 10 min in order to remove the upper layers.

One (1) mL of the CH_3_CN layers were transferred into the clean-up tubes containing 50 mg of primary-secondary amine and 150 mg of MgSO_4_. These tubes were shaken for 5 min and were centrifuged (8000 rpm) for 10 min. Portions of each tube upper layer (0.6 mL) were placed in vials for further analysis and 0.2 mL of deuterated internal standard mixture containing acenaphthene d10, chrysene, naphthalene, pyrene, and phenanthrene at 80 µg/mL were added. The reagents used for PAH extraction were from Sigma Aldrich and Merck. Twelve (12) PAHs from 16 priority PAHs (Naphthalene (Nap), Acenaphthylene (Acy), Acenaphthene (Ace), Anthracene (Ant), Phenanthrene (Phe), Fluoranthene (Flu), Pyrene (Pyr), Benzo(a)anthracene (BaA), Chrysene (Chr), Benzo(a)pyrene (BaP), Benzo(b)fluoranthene (BbF) and Benzo(k)fluoranthene (BkF)) were analyzed using a gas chromatograph-mass spectrometer (GC-MS); all of them from Agilent Technologies.

The gas chromatograph system was used in the selected ion monitoring mode based on the use of one ion. The identification of compounds was based on their qualifier ions’ times of retention. Chromatographic separations were conducted using a HP-5MS (5% Phenyl Methyl Siloxane) fused silica capillary column (30 m long × 0.25 mm internal diameter × 0.25μm film thickness). The operation temperature program of the GC oven started at 70 °C, which was held for 3 min and was increased to 240 °C with 20 °C/min, and then to 310 °C with 5 °C/min. The injector temperature was 300 °C while the transfer line temperature was 280 °C. Hence, 2.5 μL of acetonitrile extracts were injected in splitless mode. The carrier gas was helium (1.0 mL/min of flow rate). Ionization voltage of 70 eV, acquisition mass range of 40–560 and scan time of 0.32 s were the mass spectrometer conditions. Quantitative analysis was based on the corresponding quantifier ions of each PAH molecule and previously recorded retention times. The PAH calibration standards were prepared using a certified reference standard of 2000 µg/mL, containing the 12 analyzed PAHs.

### 2.3. Quality and Accuracy Control

The method’s quality and accuracy were checked by analyzing duplicates of selected sample and procedural blank reagent. These solutions were analyzed for each batch of 15 samples. The coefficients of variation of the average values of the duplicate sample were used to assess the accuracy and repeatability. The calculated coefficients of variation for analyzed pollutants were <5% and ranged between 0.11% for Pyr to 4.49% for Ant. The blank was analyzed to detect possible contamination during extraction. The PAHs molecules were not detectable in blank solutions. In addition, certified reference materials (IAEA-435; IAEA-406) were analyzed to verify the quality of the extraction procedure and reading precision. The recoveries of the 12 analyzed PAH concentrations ranged between 86.96% for Chrysene (Chr) and 107.14% for Benzo(k)fluoranthène (BkF).

### 2.4. Determination of Probable Sources of PAHs in Fish Organs

The determination of the potential origins of PAHs was carried out through several relationships between the concentrations of PAH molecules: Phe/Ant, Flu/Pyr, Ant/(Ant+Phe), Flu/(Flu+Pyr), BaA/(BaA+Chr), low molecular weight PAHs (LMW PAHs)/high molecular weight PAHs (HMW PAHs). The results are interpreted as follows: Phe/Ant and Flu/Pyr (>1: pyrogenic and <1: petrogenic), Ant/(Ant+Phe) (>0.1 pyrogenic and <0.1: petrogenic), Flu/(Flu+Pyr) (<0.4: petrogenic, 0.4–0.5: petrogenic + pyrogenic and >0.5: pyrogenic), BaA/(BaA+Chr) (<0.2 petrogenic, 0.2–0.35 petrogenic + pyrogenic and >0.35: pyrogenic) and LMW PAHs/HMW PAHs (<1: pyrogenic and >1: petrogenic) [39,40,41,42]. The LMW PAHs detected are composed of Nap, Ant, and Phe and the HMW PAHs are composed of Flu, Pyr, BaA, and Chr.

### 2.5. Statistical Analysis

Analysis of variance followed by the Newman–Keuls test were applied to the data to determine the variability in mean PAH levels between fish organs. Mean values are significantly different when *p* < 0.05. Spearman’s correlation test was performed to determine the relationship between total lengths and total weights of fish. In addition, Student’s *t* test was performed to compare the average contents of PAHs having low molecular weight (LMW PAHs) and high molecular weight (HMW PAHs). Principal component analysis (PCA) was performed to determine the overall interrelationships between individual PAHs and their distribution in organs. Statistical analyses were performed using STATISTICA 6.1 software.

### 2.6. Human Health Risk Assessment

The dietary PAHs exposures for consumers were calculated using mean concentration of each PAHs in fish’s muscles and in all organs analyzed (muscle, gill, and liver). In addition, two types of peoples were considered (adults and children). The estimated daily intake (EDI) was determined according to the following equation [43,44]:

$$\mathrm{EDI}=\frac{C\times Q\times F}{\mathrm{BW}}$$

where EDI = estimated daily intake (mg/kg/day); C = concentration of PAHs in fish’s tissues (mg/kg); Q = daily quantity of fish ingested (kg/day). These quantities are 166.75 g/day of wet weight (g/d ww) for adults and 110.25 g/d ww for children [45]; F = exposure frequency (day/year); BW = body weight (kg). The average body weights of the local population were 67.64 kg for adults of 22–60 years old and 29.40 kg for children of 3–16 years old [46,47]. The fish consumption frequency is considered as equal to one (365 days/year).

The risk of non-carcinogenic effects was expressed by the calculation of the hazard quotient (HQ). The HQ were calculated using the following equations [48,49]:

$$\mathrm{HQ}=\frac{\mathrm{EDI}\times \mathrm{ED}}{{\mathrm{RfD}}_{o}\times T_{m}}$$

where RfD_o_ is the oral reference dose (mg/kg/day). ED = exposure duration (year); T_m_ = total life duration (year). For non-carcinogenic effects, the ED is equal to T_m_. If HQ < 1, toxic effect is less probable; if HQ > 1, toxic effect cannot be excluded. In order to determine the additive and/or iterative effects of PAHs, total hazard quotient (THQ) was calculated using the following equation [50,51]:

$$\mathrm{THQ}=\overset{n}{\underset{i=1}{\sum }}{\mathrm{HQ}}_{i}$$

The cancer risk (CR) is the probability of an individual to develop cancer, due to the exposure over a lifetime [52]. The cancer risk was calculated by the following formula [35,50]:

$$\mathrm{CR}=\mathrm{EDI}\times {\mathrm{CSF}}_{o}\times \frac{\mathrm{ED}}{T_{m}}$$

where CSF_o_ is the oral cancer slope factor (mg/kg/day)^−1^.

For carcinogen effects, the ED is defined as equal to 30 years and the Tm is 70 years according to USEPA (USEPA, 1991). The CR interpretation is as follow: CR < 10^−6^ is negligible; 10^−6^ < CR < 10^−4^ is acceptable et CR > 10^−4^ is unacceptable [50,53,54]. Oral reference doses (RfDo) and oral cancer slope factor (CSFo) are depicted in Table 1.

## 3. Results

### 3.1. Biometrics of Fish

**Figure 2:**
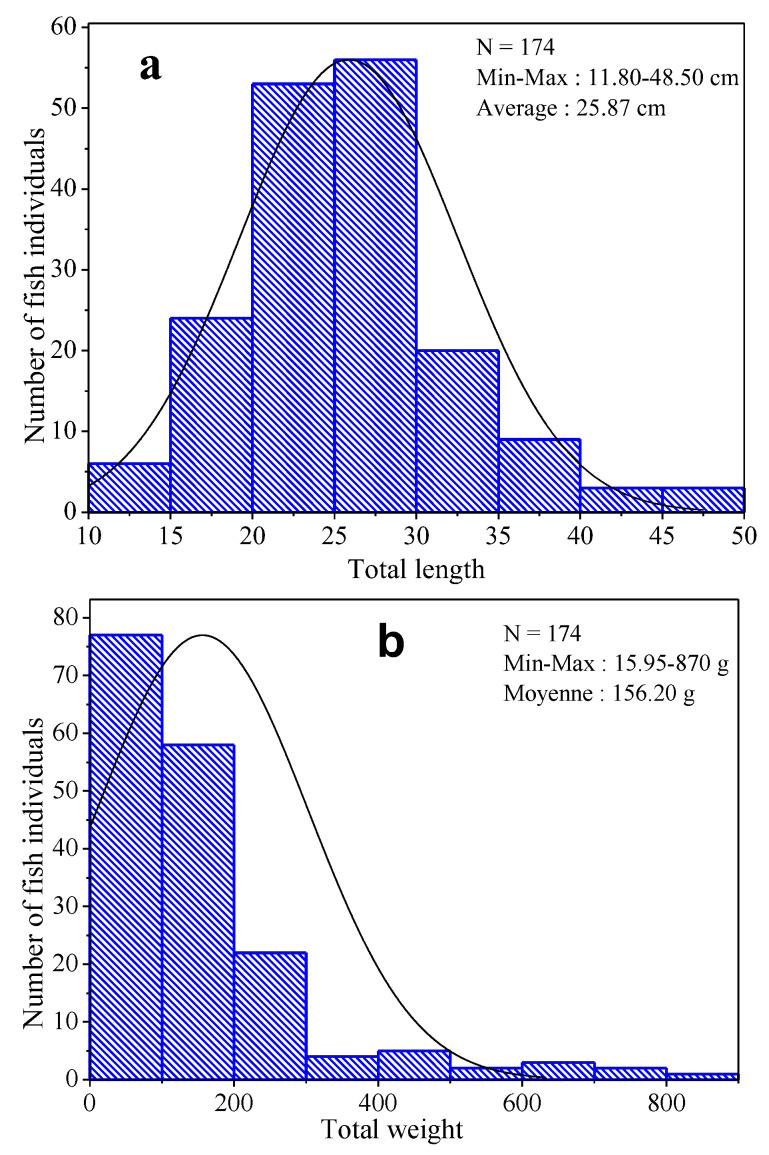
Frequency histograms of fish total lengths (**a**) and total weights (**b**).

The results of the biometric measurements of the fish (Figure 2a,b) show that the total lengths of the fish studied vary from 11.80 to 48.50 cm with an average of 25.87 ± 6.65 cm (Figure 2a). In addition, their total weights are between 15.95 and 870 g with an average of 156.20 ± 146.62 g (Figure 2b). Spearman’s correlation analysis between the two data sets showed that there is a strong and positive correlation between total lengths and total weights of fish (r = 0.92; *p* < 0.0001).

### 3.2. Polycyclic Aromatic Hydrocarbons (PAHs) in Fish Organs

Among the 12 priority PAHs analyzed, it can be noted from the results presented in Table 2 that only seven PAHs (Nap, Ant, Phe, Flu, Pyr, BaA, Chr) were detected in most of the samples. The remains of PAHs (Acy, Ace, BaP, BbF, BkF) were not detected in all samples analyzed. The mean concentrations of PAHs obtained in the muscles vary from 1.42 ± 0.37 µg/kg for fluoranthene (Flu) to 6.69 ± 4.15 µg/kg for naphthalene (Nap). Phenanthrene (Phe) exhibited the highest concentration of PAHs in muscles (30.7 µg/kg). In the gills, the average PAH contents vary from 1.43 ± 0.39 µg/kg for pyrene (Pyr) to 5.41 ± 3.91 µg/kg for Phe, which at the same time has the highest concentration PAHs in the gills (18 µg/kg). The average PAH levels in the liver are between 2.63 ± 0.90 µg/kg for Pyr and 41.71 ± 21.14 µg/kg for Nap, which also has the maximum value of the PAH levels recorded (94.7 µg/kg) in the liver.

The concentrations of total PAHs (tPAHs) vary from 5.24 to 48.4 µg/kg with an average of 14.51 ± 8.95 µg/kg in the muscles. In the gills, they are between 5.90 and 28.20 µg/kg with an average of 14.90 ± 5.19 µg/kg while in the livers, they vary from 43.20 to 149 µg/kg with an average of 80.74 ± 27.08 µg/kg. The increasing order of accumulation of tPAHs in *C. nigrodigitatus* organs is as follows: muscle < gill < liver (Table 1).

**Figure 3:**
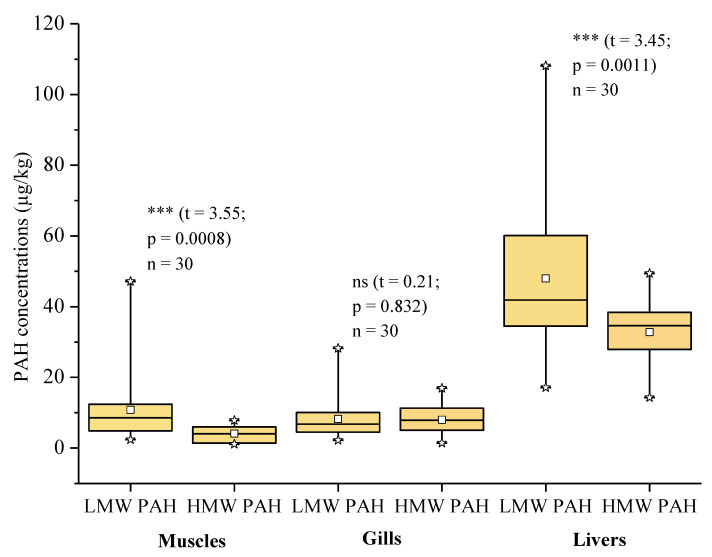
Boxplot diagrams of LMW and HMW PAHs concentrations in fish organs. Note: ***: Significant test (*p* < 0.05), ns: non-significant test (*p* > 0.05).

The concentrations of low molecular weight PAHs (LMW PAHs) vary from 2.4 to 47.1 µg/kg in the muscles, from 2.2 to 28.2 µg/kg in the gills and from 17.1 to 108.1 µg/kg in the livers. The concentrations of high molecular weight PAHs (HMW PAHs) are between nd and 7.8 µg/kg in muscles, between nd and 16.9 µg/kg in the gills and between 14.3 and 49.4 µg/kg in the livers. The *t* test indicates that the average concentrations of LMW PAHs (10.82 µg/kg) are significantly higher than those of HMW PAHs (4.10 µg/kg) for muscles (t = 3.55; *p* = 0.0008) and for livers (t = 3.45; *p* = 0.0011), the averages of which are respectively 47.97 µg/kg and 32.77 µg/kg. This difference is not significant (*p* > 0.05) for the gills. However, it is noted that the average concentration of LMW PAHs (8.25 µg/kg) is slightly higher than that of HMW PAHs (7.97 µg/kg) (Figure 3). The increasing order of accumulation of HMW PAHs in organs is as follows: muscle < gill < liver while that of LMW PAHs is as follows: gill < muscle < liver.

Although BaP molecules were not detected in all of the samples, the sum of the four PAHs (PAH4) composed of BaP, BaA, BbF, and Chr according to the European Commission evaluations ranged between 1.3 and 7.3 µg/kg for muscles, 2.3 and 13.9 µg/kg for gills, and 10.9 to 45 µg/kg for livers.

**Figure 4:**
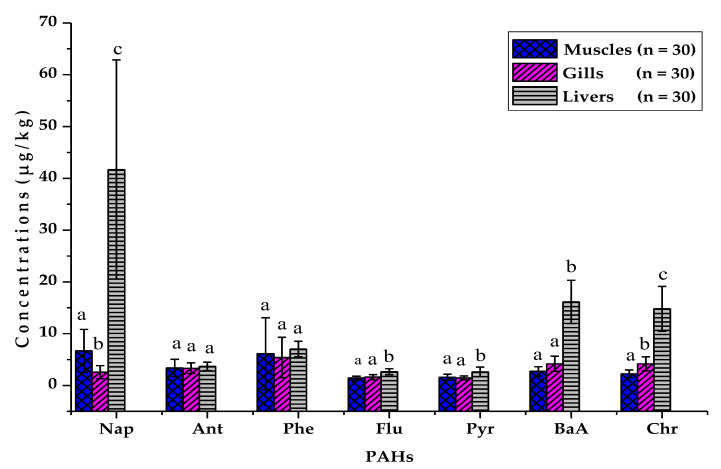
Comparison of PAH concentrations between fish organs. Note: For each element, the organs bearing the same letters are statistically homogeneous unlike those bearing different letters (ANOVA followed by Newman-Keuls test).

Analysis of variance (ANOVA) showed a significant variation (6.41 ≤ F ≤ 156.54; 0.00001 < *p* < 0.01) in PAH levels between organs except for anthracene and phenanthrene. The Newman–Keuls test indicates that the PAH contents of muscles and gills are globally homogeneous while the PAH contents obtained in the livers are for the most part different from and higher than those of the muscles and gills (Figure 4). The order of organ contamination is as follows: muscle < gills < liver for Flu, BaA, Chr and gills < muscle < liver for Nap, Ant, Phe, and Pyr (Figure 4).

### 3.3. Principal Components Analysis of PAHs Concentrations in Fish

**Figure 5:**
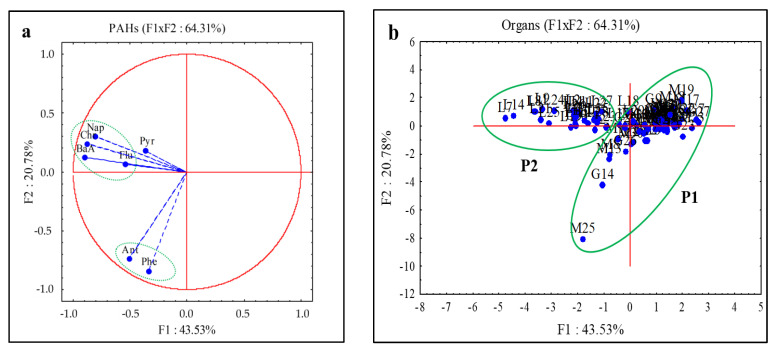
Projection of variables (PAHs) (**a**) and observations (Organs) (**b**) in the factorial plane F1 × F2 (M: muscle, G: gills, L: liver).

The eigenvalues, the total variances expressed, and the correlation coefficients between the variables and the factorial axes are presented in Table 3. This table shows that the first three factors represent 77.36% of the total variance, including F1: 43.53 %, F2: 20.78%, and F3: 13.05% (Table 2). The F1 axis is negatively defined by BaA, Chr, Nap, and Flu according to the correlation coefficients presented in Table 3 and the projection of the variables in the F1xF2 plane. This axis shows, from right to left, an increasing gradient of contamination in PAHs of high molecular weight for the most part (Figure 5a). The F2 axis is strongly defined in its negative part by Phe (−0.84) and Ant (−0.74) representing, from top to bottom, an increasing gradient of contamination in Phe and Ant which are LMW PAHs (Table 3; Figure 5a). The F3 axis is strongly determined (0.90) in its positive part by Pyr (Table 3).

The projection of observations (organs) in the F1xF2 plane made it possible to discriminate two groups of organs according to their level of contamination. The group (P1), composed of muscles and gills is characterized by a lower contamination of PAHs with the exception of samples M25 (muscle 25) and G14 (gills 14) for phenanthrene and anthracene. The second group (P2) is composed of livers which are generally characterized by a higher concentration of PAHs. The result is an increasing gradient in the accumulation of PAHs from the muscles to the livers (Figure 5b).

**Figure 6:**
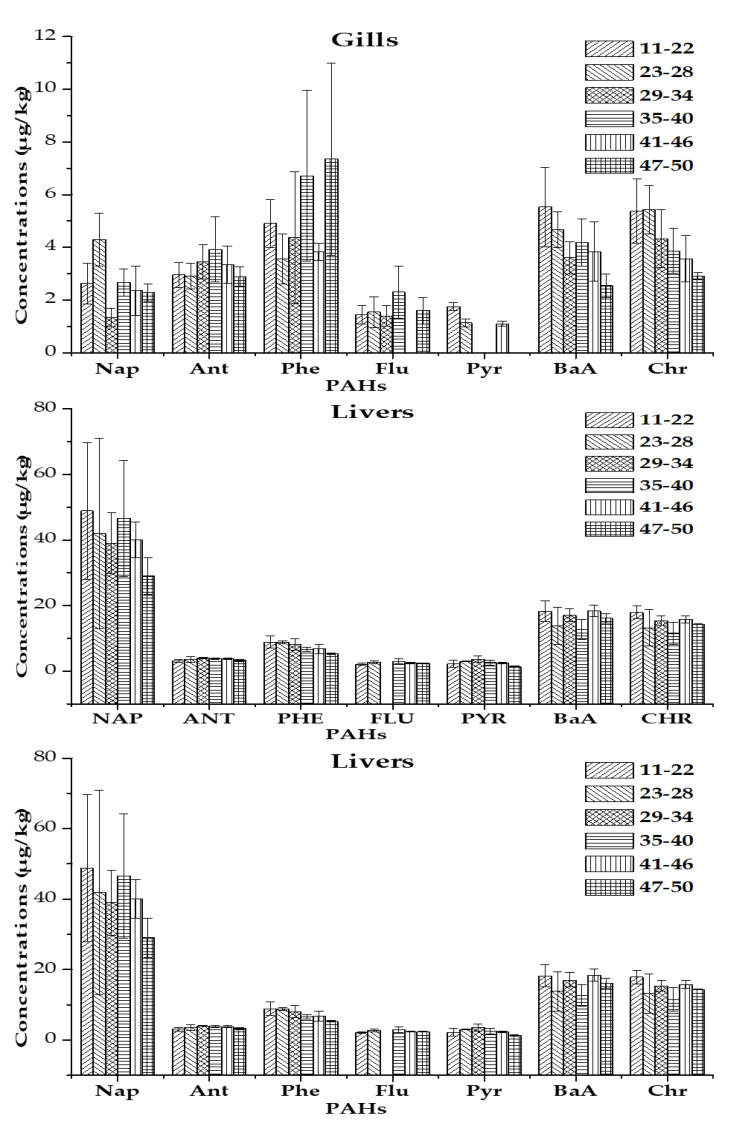
Variation of PAH contents according to size classes of fish.

The PAH contents of the organs varied irregularly according to the size classes. However, an overall trend of decreasing levels was observed with increasing fish size classes for Nap and Pyr in muscles, for Pyr, BaA, and Chr in gills, and for Nap and Phe in livers (Figure 6).

### 3.4. Probable Source of PAHs in Fish Organs

The results of the different average ratios (Table 4) indicate that the PAHs present in the organs of fish come from two sources (pyrogenic and petrogenic). Although the mean ratio (LMW PAHs/HMW PAHs) is greater than 1 in the gills, indicating a petrogenic source, 64% of individuals presented ratios lower than 1. These PAHs are therefore mainly of pyrogenic origin linked to the combustion of organic matter of origin, fossil or synthetic (fuel, fuel, plastic, rubber, vegetation etc.).

### 3.5. Human Health Risks Assessment Associated with Fish Consumption

The hazard quotients (HQ) calculated for adults and children as a function of two forms of consumption are presented in Table 5. The results show that these HQ are all less than 1 for both groups of exposure and two possible forms of fish consumption. In addition, all of the total hazard quotients (THQ) are less than 1 and range from 1.63 × 10^−3^ for the consumption of muscle alone in adults to 4.50 × 10^−3^ for the consumption of all three organs in children. However, it has been shown that children are more exposed than adults with HQ that are higher than those of adults regardless of the exposure scenario.

The cancer risk (CR) results for non-threshold or carcinogenic effects are presented in Table 6. This indicates that CR are mostly negligible (<10^−6^) and acceptable (10^−6^ < CR <10^−4^) for a few. The latter vary from 1.29 × 10^−6^ in children for the consumption of muscles alone to 3.28 × 10^−6^ in children for the consumption of the combined three organs.

## 4. Discussion

The average length (25.87 cm) obtained indicates that the *C. nigrodigitatus* individuals studied are mostly mature [61,62,63]. These sizes are broadly similar to those observed in *C. nogrodigitatus* individuals in the West African sub-region [64,65,66,67]. However, these sizes are larger than those reported by Atobatele and Ugwumba [68] in Aiba Reservoir in Nigeria (9.8–25.6 cm) while longer individuals have been observed by Andem et al. [69] under the Itu bridge in Nigeria (9–109 cm). In addition, the strong and positive correlation obtained between the lengths and the weights of the fish is in agreement with the results obtained by Lawal et al. [67] in the same species in the Epe lagoon in Nigeria (r = 0.868) and in *Chrysichthys furcatus* from the Cross River in Nigeria (r = 0.97) by Irom et al. [70].

The different routes of exposure of fish to contaminants in aquatic environments are mainly the direct bioconcentration of molecules dissolved in water through their gills and skin, as well as the ingestion of contaminated food and sediment particles. The rate of bioaccumulation may depend on the food preferences, habitats, and the trophic level of fish [10,71,72]. The comparison of the results with other work is presented in Table 7. Indeed, the concentrations of tPAHs observed in this study are lower than those obtained in fish from the Ghanaian coast [73] and from Ogun and Eleyele Rivers in Nigeria [19]. In addition, the PAH concentrations recorded in the muscles of fish from the coastal waters of Benin are generally higher than those of the present study [74]. However, the results from this study are higher than those obtained in *Tilapia guineensis* and *Liza falcipinnis* in Nigeria [72] and in catfish from Hong Kong markets [71] (Table 7).

The maximal levels of PAHs set and reviewed by European Commission (Commission Regulation 1255/2020) in fish concern individual BaP molecule (5 µg/kg) as the main marker for PAHs in food and the sum of four PAHs molecules (PAH4) composed of BaP, BaA, BbF, and Chr (30 µg/kg) [77]. In the present study, BaP molecules were not detected in all of the samples analyzed. However, 67% of the analyzed liver samples presented PAH4 concentrations higher than the permissible level.

The highest average concentrations of PAHs having low molecular weight (LMW PAHs) recorded in the three fish organs are in agreement with other studies [1,26,74,76,78]. This strong accumulation of LMW PAHs may be due to their high solubility in water, their high bioavailability, and a high metabolism of HMW PAHs [1,26]. The high average Nap (LMW PAHs) concentrations observed in muscles (6.69 µg/kg) and livers (41.71 µg/kg) could be explained by the high solubility of Nap in water and its high bioavailability rendering it strongly absorbed in the water environment [1,79]. Indeed, due to their high solubility, LMW PAHs have an accumulation rate three times higher than HMW PAHs one in aquatic organisms [80]. These results agree with those obtained in other previous studies [1,73,79]. However, bioaccumulation is controlled by several factors, such as the duration of exposure, the quality and mode of feeding, the species, the nature of the molecule, the physicochemical quality of the medium (temperature, salinity, pH, etc.), as well as the absorption and removal rates of contaminants [79]. The abundance of Nap and Phe could be linked to the release of used fuel and motor oils into the wild from the phosphate processing plant and quarry machinery. In addition, automobile exhaust gases can be noted as a result of atmospheric depositions. Indeed, it is known that PAHs with two and three aromatic rings, such as Nap and Phe, are characteristic of PAHs of petrogenic origin [71,81]. This abundance of Nap and Phe has also been observed in other studies [1,71,73].

The double pyrogenic origin of PAHs observed in this study is in agreement with other studies. This may be due to the complexity of the parameters that influence the PAHs distribution in the environment [39,40]. However, attention should be paid to the petrogenic origin of PAHs in fish. Indeed, the high concentrations of LMW PAHs observed could be linked to their high solubility in water. This renders them more bioavailable, associated to the fact that HMW PAHs can be easily removed from fishes [73,82]. In addition, it is known that in tropical environments, Nap and Phe can also have a biological source [83]. The main entry routes for PAHs into the lagoon complex can be marine intrusion, especially during low water and high tides, runoff, and rivers after leaching of mining and urban soils, as well as atmospheric depositions.

The average concentrations of tPAHs and HMW PAHs in the muscles are generally lower than those obtained in the internal organs (gill, liver). These high concentrations of HMW PAHs in the gills are thought to be due to the fact that they are better bound to particulate matter naturally retained by the gills [73]. The strong accumulation of PAHs in livers could be explained by the important physiological role that play livers in fish’s metabolism [84,85,86]. This strong accumulation by internal organs is consistent with results obtained by other studies [73,76,87]. In addition, these differences in PAHs contents in organs can be controlled by the physicochemical parameters of PAHs, lipid contents, and the metabolic capacity of each organ [76]. The overall decrease in organic pollutant content with increasing biological parameters has also been observed in other studies [1,88].

Decreases in concentrations with increasing fish sizes observed for some PAH molecules could be due to the dilution of concentrations during fish growth and low accumulation rate in old individuals. In fact, Douben [89] indicated that the accumulation of contaminants could reach a stable state after a certain age in fish. So, any further growth would dilute the existing concentrations. In addition, the oldest individuals have a more developed enzymatic system and elimination pathways (excretion or reproduction) than the youngest [45,90]. It can also be mentioned that the changes in fish diets with increasing fish sizes [29] may contribute to these decreases of concentrations since accumulation of contaminants is also linked to the composition of diet. Thus, adults that feed on less contaminated preys may be less exposed to contaminant accumulation.

The HQ and CR indicate that consumption of the fish studied does not present a risk related to PAHs on the health of consumers according to the exposure scenarios of the present study. However, increasing ingested quantities may increase risks related to PAH. These results are consistent with those obtained in some fishery products from the Persian Gulf [1]. However, the values of HQ and CR in children are always higher than in adults. This observation may be due to their low body weight, their physiological predisposition, the fragility of their organism, and their less developed enzymatic system [91,92].

## 5. Conclusions

This study provided information on the contamination of *C. nigrodigitatus* in the Lake Togo-Lagoon Aného complex. In fact, not insignificant levels were observed in the organs of fish with the highest concentrations recorded in the liver for Nap (13.5–94.7 µg/kg) and total PAH (43.20–149 µg/kg). These levels vary irregularly with the size of the fish. However, overall decreases in concentrations with increasing fish length were observed for some PAH molecules (Nap, Pyr, BaA, Phe). The PAHs present in these fish come mainly from pyrogenic sources, such as the combustion of vegetation, wood, plastics, rubbers, coals, and fossil fuels. It emerges that the consumption of these fish does not present any health dangers if the exposure scenarios of this study are respected (HQ < 1 and CR < 10^−4^). However, it is urgent to take measures for the efficient management of these resources in order to limit or avoid any public health problem.

## Figures and Tables

**Table 1:** RfDo for non-carcinogenic effects and CSFo for carcinogenic effects.

PAHs	RfDo: Oral Reference Dose (mg/kg/d)		CSFo: Oral Cancer Slope Factor (mg/kg/d)^−1^	
	Values	Agency and Years	Values	Agency and Years
Nap	2 × 10^−2^	US EPA/1998 [55]	1.2 × 10^−1^	OEHHA/2005 [56]
Ant	4 × 10^−2^	RIVM/2001 [57]	1 × 10^−2^	INERIS/2018 [58]
Phe	4 × 10^−2^	RIVM/2001 [57]	1 × 10^−3^	INERIS/2018 [58]
Flu	4 × 10^−2^	US EPA/1990 [59]	5 × 10^−2^	RIVM/2001 [57]
Pyr	3 × 10^−2^	Canada/1993 [60]	5 × 10^−1^	RIVM/2001 [57]
BaA	-	-	10^−1^	INERIS/2018 [58]
Chr	-	-	10^−2^	INERIS/2018 [58]

**Table 2:** Statistical parameters of polycyclic aromatic hydrocarbons contents in fish organs.

Statistical Parameters	PAH Concentrations [µg/kg of Wet Weight (µg/kg w.w.)]												tPAHs
	Nap	Acy	Ace	Ant	Phe	Flu	Pyr	BaA	Chr	BaP	BbF	BkF	
Muscles (n = 30)													
Min	<1	-	-	1.1	1	<1	<1	<1	<1	-	-	-	5.24
Max	16.4	-	-	9.2	30.7	2	2.4	4.5	3.5	-	-	-	48.40
Average	6.69	nd	nd	3.38	6.12	1.42	1.59	2.72	2.24	nd	nd	nd	14.51
SD	4.15	-	-	1.66	6.94	0.37	0.58	0.89	0.74	-	-	-	8.95
Gills (n = 30)													
Min	<1	-	-	1.7	1.8	<1	<1	<1	2.1	-	-	-	5.90
Max	5.4	-	-	6.8	18	2.3	1.9	7.1	6.8	-	-	-	28.20
Average	2.56	nd	nd	3.31	5.41	1.60	1.43	4.19	4.16	nd	nd	nd	14.90
SD	1.26	-	-	1.04	3.91	0.50	0.39	1.45	1.36	-	-	-	5.19
Livers (n = 30)													
Min	13.5	-	-	2.3	<1	<1	<1	6.3	4.6	-	-	-	43.20
Max	94.7	-	-	5.2	9.3	3.7	4.6	23.2	22.9	-	-	-	149.00
Average	41.71	nd	nd	3.69	7.02	2.67	2.63	16.14	14.78	nd	nd	nd	80.74
SD	21.14	-	-	0.81	1.51	0.55	0.90	4.18	4.33	-	-	-	27.08
Average of organs	16.99	nd	nd	3.46	6.18	1.90	1.88	7.68	7.06	nd	nd	nd	45.15

nd: not detected; ww: wet weight.

**Table 3:** Eigenvalues, total variances and correlation between variables and axes.

	F1	F2	F3
BaA	−0.90	0.12	−0.08
Chr	−0.88	0.24	−0.17
Nap	−0.81	0.30	−0.01
Flu	−0.55	0.07	−0.25
Phe	−0.34	−0.84	0.11
Ant	−0.51	−0.74	0.00
Pyr	−0.37	0.18	0.90
Eigenvalues	3.05	1.45	0.91
Total variance explained	43.53	20.78	13.05
Cumulative total variance	43.53	64.31	77.36

**Table 4:** Results of the various PAH concentration ratios and the probable source.

	Average Ratios of PAH Concentrations					
	Phe/Ant	Flu/Pyr	Ant/(Ant+Phe)	Flu/(Flu+Pyr)	BaA/(BaA+Chr)	LMW PAHs/HMW PAHs
Muscles (n = 30)						
Value	1.71	0.81	0.66	0.91	0.59	4.66
Source	Pyrog.	Petrog.	Pyrog.	Pyrog.	Pyrog.	Petrog.
Gills (n = 30)						
Value	1.37	0.90	0.69	0.87	0.53	1.26
Source	Pyrog.	Petrog.	Pyrog.	Pyrog.	Pyrog.	Petrog.
Livers (n = 30)						
Value	1.97	0.82	0.76	0.84	0.52	1.52
Source	Pyrog.	Petrog.	Pyrog.	Pyrog.	Pyrog.	Petrog.

Note: Pyrog. = pyrogenic, Petrog. = petrogenic.

**Table 5:** Hazard quotient (HQ) for non-carcinogenic effect.

PAHs	Hazard Quotient (HQ)			
	Muscles		Combined Three Organs	
	Adults	Children	Adults	Children
Nap	8.25 × 10^−4^	1.25 × 10^−3^	2.09 × 10^−3^	3.19 × 10^−3^
Ant	2.08 × 10^−4^	3.17 × 10^−4^	2.13 × 10^−4^	3.24 × 10^−4^
Phe	3.77 × 10^−4^	5.74 × 10^−4^	3.81 × 10^−4^	5.80 × 10^−4^
Flu	8.75 × 10^−5^	1.33 × 10^−4^	1.17 × 10^−4^	1.78 × 10^−4^
Pyr	1.31 × 10^−4^	1.99 × 10^−4^	1.55 × 10^−4^	2.35 × 10^−4^
THQ	1.63 × 10^−3^	2.48 × 10^−3^	2.96 × 10^−3^	4.50 × 10^−3^

**Table 6:** The cancer risk (CR) for carcinogenic effect.

PAHs	Cancer risk (CR)			
	Muscles		Combined Three Organs	
	Adults	Children	Adults	Children
Nap	8.48 × 10^−7^	1.29 × 10^−6^	2.15 × 10^−6^	3.28 × 10^−6^
Ant	3.57 × 10^−8^	5.43 × 10^−8^	3.66 × 10^−8^	5.56 × 10^−8^
Phe	6.47 × 10^−9^	9.84 × 10^−9^	6.53 × 10^−9^	9.94 × 10^−9^
Flu	7.50 × 10^−8^	1.14 × 10^−7^	1.00 × 10^−7^	1.52 × 10^−7^
Pyr	8.40 × 10^−7^	1.28 × 10^−6^	9.95 × 10^−7^	1.51 × 10^−6^
BaA	2.87 × 10^−7^	4.37 × 10^−7^	8.12 × 10^−7^	1.23 × 10^−6^
Chr	2.37 × 10^−8^	3.60 × 10^−8^	7.46 × 10^−8^	1.13 × 10^−7^

**Table 7:** Comparison of the concentrations of the present study with those of the literature.

Fish Organs	PAHs (µg/kg)													Reference
	Nap	Acy	Ace	Ant	Phe	Flu	Pyr	BaA	Chr	BaP	BbF	BkF	HAP t	
Muscle	3.85	1.56	-	-	0.3	5.19	-	-	-	-	-	-	10.9	[1]
Muscle	0.18	0.38	17.20	10.93	41.71	8.79	10.56	-	7.20	0.15	18.16	9.43	118.34	[40]
Muscle	4.60	0.60	0.98	1.56	2.99	1.91	1.45	0.35	0.73	n.d.	n.d.	n.d.	15.15	[71]
Muscle	17.68	2.28	2.90	2.09	2.00	2.90	3.69	11.87	2.90	1.85	-	nd	50.12	[39]
Muscle	0.11	7.21	3.29	0.29	0.16	0.27	0.95	3.82	0.19	1.18	58.83	0.07	71.52	[72]
Muscle	nd	0.23	1.07	0.61	0.52	0.16	0.20	0.48	0.03	2.99	53.07	0.03	58.30	
Muscle	-	-	-	-	-	-	-	-	-	-	-	-	165.22	[73]
Gill	-	-	-	-	-	-	-	-	-	-	-	-	238.75	
Muscle	2.35	2.15	2.40	2.55	1.80	3.50	1.45	1.20	1.60	0.70	1.40	4.00	25.10	[75]
Muscle	1.35	1.09	1.07	1.26	0.71	1.08	1.99	3.085	3.91	12.34	-	-	27.90	[19]
Gill	1.09	2.66	1.65	1.39	0.90	1.88	1.89	3.99	10.86	6.97	-	-	33.31	
Liver	1.70	1.89	1.37	1.03	0.74	1.62	1.76	3.57	3.89	3.79	-	-	21.38	
Muscle	1.55	0.23	0.14	4.54	13.36	0.53	3.17	0.02	0.05	0.00	0.03	0.01	23.63	[76]
Gill	2.05	0.37	0.15	6.92	6.35	0.85	1.18	0.18	0.64	0.09	0.11	0.04	18.91	
Liver	2.38	0.62	0.22	1.73	6.01	5.05	2.10	0.28	0.20	0.18	0.30	0.12	19.18	
Muscle	-	-	-	2.66	6.85	1.47	2.13	1.15	2.63	1.75	0.78	0.35	19.76	[74]
Muscle	6.69	nd	nd	3.38	6.12	1.42	1.59	2.72	2.24	nd	nd	nd	14.51	Present study
Gill	2.56	nd	nd	3.31	5.41	1.6	1.43	4.19	4.16	nd	nd	nd	14.90	
Liver	41.71	nd	nd	3.69	7.02	2.67	2.63	16.14	14.78	nd	nd	nd	80.74	

Note: nd = not detected.

## Data Availability

Not applicable.
